# Effect of micro-plastic particles on coral reef foraminifera

**DOI:** 10.1038/s41598-024-63208-3

**Published:** 2024-05-30

**Authors:** Alexander Zientek, Michael Schagerl, Matthias Nagy, Wolfgang Wanek, Petra Heinz, Sameh S. Ali, Michael Lintner

**Affiliations:** 1 Department of Palaeontology, University of Vienna, Vienna, Austria; 2 Department of Functional and Evolutionary Ecology, University of Vienna, Vienna, Austria; 3 Department of Microbiology and Ecosystem Science, University of Vienna, Vienna, Austria; 4 Botany Department, Faculty of Science, Tanta University, Tanta, 31527 Egypt; 5 ING PAN - Institute of Geological Sciences, Polish Academy of Sciences, Research Centre in Krakow, Krakow, Poland

**Keywords:** Micro-plastic uptake, Marine pollution, Laboratory experiments, Foraminifera, Ecology, Environmental sciences, Ocean sciences

## Abstract

Foraminifera are single-celled protists which are important mediators of the marine carbon cycle. In our study, we explored the potential impact of polystyrene (PS) microplastic particles on two symbiont-bearing large benthic foraminifera species—*Heterostegina depressa* and *Amphistegina lobifera*—over a period of three weeks, employing three different approaches: investigating (1) stable isotope (SI) incorporation—via ^13^C- and ^15^N-labelled substrates—of the foraminifera to assess their metabolic activity, (2) photosynthetic efficiency of the symbiotic diatoms using imaging PAM fluorometry, and (3) microscopic enumeration of accumulation of PS microplastic particles inside the foraminiferal test. The active feeder *A. lobifera* incorporated significantly more PS particles inside the cytoplasm than the non-feeding *H. depressa,* the latter accumulating the beads on the test surface. Photosynthetic area of the symbionts tended to decrease in the presence of microplastic particles in both species, suggesting that the foraminiferal host cells started to digest their diatom symbionts. Compared to the control, the presence of microplastic particles lead to reduced SI uptake in *A. lobifera*, which indicates inhibition of inorganic carbon and nitrogen assimilation. Competition for particulate food uptake was demonstrated between algae and microplastic particles of similar size. Based on our results, both species seem to be sensitive to microplastic pollution, with non-feeding *H. depressa* being more strongly affected.

## Introduction

### Plastic pollution in the marine environment

In 2021, an estimated 390 million metric tons of plastics was produced worldwide (Statista Research Department, 2023). Around 10% of the produced plastics are finally discharged into the ocean (Statista Research Department, 2023). About 80% of the plastics found in the ocean are of terrestrial origin and are transported to the sea by rivers^1^. Plastics can sink with time which makes them also omnipresent in seafloor sediments, even at great depths^2^. Some studies have shown that PE and PP are abundant in sediments, while PS is less abundant^2^. Since PS particles are derived from Styrofoam used in fishing gear and fish preservation containers, they are a more potential risk in shallow waters^3^. In shallow water like in the Kiel Fjord an average pollution of 1.8–4.5 microplastic particles (fibres plus fragments) per kg of dry sediment was found^4^. Between March 2018 and March 2019, Ory et al.^5^ monitored microplastics concentrations in eight different stations and found a stable entry of microplastic from 0.04 particles/m^3^ water. Plastic particles are also present in coral reefs. In 2022 the micro- and nano plastic particles in Palau’s coral reefs have been investigated and a maximum number of 32 microparticles and 71 nanoparticles per dry weight sediment was found^6^.

In the ocean plastic materials are mechanically or chemically fragmented, but also broke down due to biological weathering into smaller pieces^1,7^. They pose a serious risk to the environment because smaller particles more easily distributed across large distances and are more likely to be taken up by marine organisms. Moreover, they are much harder to detect and to remove from the environment^8^. Plastic particles are categorized according to their size, but no common classification does yet exist. More than 90% of all plastic waste in the ocean consists of particles smaller than 5 mm^9^ and particles smaller than 1 mm make up 65% of all plastic debris^10^. Because of continued fragmentation of macroplastic and microplastic, the extent of microplastic contamination would still increase over time even if no additional plastic would enter the ocean.

The most common plastic materials found in the ocean are composed of polyethylene (PE), polypropylene (PP) and polystyrene (PS)^8,11^. In this study we will focus on PS particles since they are often found in marine sediments due to their usage in aquaculture or their input through urban cities^12^. Technically, all those materials are biochemically inert and none of them is inherently toxic^13^. It is however the additives that are most problematic since they are used in high concentrations, are often easily leached from the polymers and for the most part are lipophilic which enables them to penetrate cell membranes^8^. Whether microplastic particles can be taken up by marine organisms has been tested in numerous studies in the past three decades. Bhattacharya et al.^14^ for example observed the uptake of PS beads by two algae species, *Chlorella* sp. and *Scenedesmus* sp. Browne et al.^15^ showed the accumulation of plastic beads in the mussel *Mytilus edulis*. Microplastic particle ingestion has also been proven for seabirds^16^, sea turtles^17^, whales^18^ and fish^19^. These are just a few examples. It has also been proven in various studies that ingested microplastic particles can be transferred through the food web and from organism to organism^20^.

### Pollution effects on marine foraminifera

Microplastic pollution and other anthropogenic pollutants are widespread and have a significant impact on organisms in the lower trophic levels that support marine ecosystems, and their effects are creeping up on foraminifera, important carbonate producers in the ocean. They are widely recognized in the scientific community and are often compared to corals in terms of their importance for marine ecosystems and the marine carbon cycle, due to the calcification of their endoskeleton (“tests”) and their high productivity through algal symbionts. The reef regions (while only constituting about 0.2% of oceanic surface) are responsible for one sixth of the ocean’s carbonate production. Corals and their symbionts are responsible for most of this production, but about 5% of the reef carbonate production (about 40 million tons annually) is contributed by foraminifera, and 80% of that by large benthic foraminifera (LBF)^21^. About 30% of the atmospheric CO_2_ released by humans has been absorbed by the oceans over the last 200 years^22^ and foraminifera hereby made an important contribution. The biomineralization process of CaCO_3_ by foraminifera leads to a decrease of CO_2_ concentration in ocean water while the carbon bound in the tests of these foraminifera sinks to be buried in marine sediments^23^, therefore the impact of plastics may also affects carbon fixation and photosynthetic activity related to the productivity of calcium carbonate. It has been suggested that research on microplastics for foraminifera should also be encouraged because of their importance as blue carbon producers^24^. Although, CO_2_ fixation by calcifying organisms is till controversial, since the net balance between carbonate production (CO_2_ release) and dissolution (CO_2_ sink) in calcifying organisms remains unexplored in general^25^.

The effects of various heavy metals and chemicals on foraminifera have been studied extensively, but there are few studies on microplastics, despite their importance as a carbon fixer for foraminifera.

Although there are many studies on pollutant responses of foraminifera, research on the impact of microplastics on foraminifera is relatively scarce compared to the magnitude of plastic pollution present in the oceans^24^. Only a few studies have been conducted so far on this topic. Ciacci et al.^26^ investigated the uptake and the effect of PS nanoparticles on *Ammonia parkinsoniana*. After 24 h exposure, they were able to locate microplastic particles in the foraminifera and noticed that the mitochondria of treated individuals were swollen and degraded. Moreover, the production of reactive oxygen species (ROS) was increased. The authors assumed that the most likely route of the particles entering the organism was through phagocytosis similar to the entry path of food or detritus^26^. The uptake of microplastic particles by foraminifera was studied in detail by Grefstad^13^, using amendment of fluorescent PS-beads of different sizes (0.5 μm, 1 μm, 6 μm) into sediment for up to four weeks and sampling and assessing microplastic uptake by living foraminifera. Of all identified 41 species (*H. depressa* and *A. lobifera* were not detected), 17 species had incorporated microplastic particles after six hours and 21 species did so after four weeks^13^. Most foraminifera species did not differentiate between microplastic particle size. Birarda et al.^27^ used FTIR microscopy to identify plastic particles in foraminifera collected from plastic bag surfaces on the sea floor and found incorporation of microplastics of respective bag composition. The authors included in vitro experiments of *Rosalina globularis* treated with the commonly used plasticiser Di(2-ethylhexyl)-phthalate (DEHP) and confirmed incorporation into the cytoplasm^27^. These results highlight that foraminifera could potentially be harmed by plasticisers and other compounds released from plastic waste even if the microplastic itself is not taken up. Langlet et al.^28^ tested polypropylene leachates on *Haynesina germanica* at environmentally realistic and chronic concentrations to explore its potential influence on locomotion and/or metabolism. They however were not able to detect significant effects, suggesting that benthic foraminifera might be more resistant to some pollutants than marine metazoans.

The question whether foraminifera can differentiate between microplastic and food particles was recently investigated by Joppien et al.^29^. The authors compared the number of interactions by *A. lobifera gibbosa* with polyethylene particles to the number of interactions with nauplii larvae of *Artemia* sp. They found that the foraminifera exhibited a strong preference for the larvae^29^. The applied plastic particles had a similar size than the nauplii larvae but they were too large (150–300 μm) to be actively ingested. Nonetheless, the experiment shows that *A. lobifera gibbosa* can differentiate between plastic and food.

It is undeniable at this point, that microplastics are encountered by virtually any marine organism, regardless of habitat and size, and that it can have potentially harmful effects on them. This study aims to investigate the uptake of microplastic particles (2 µm size) by two LBF species, *H. depressa* and *A. lobifera*, as well as the effect of those particles on the health of the foraminifera. *H. depressa* is known to be exclusively dependent on photosymbionts while *A. lobifera* uses a combination of photosymbiosis and particle feeding for its nutritional demand^30^. Based on the different metabolic lifestyles, we proposed that *A. lobifera* will take up more microplastic particles than *H. depressa* under the same environmental conditions. We further assumed that the uptake and accumulation of microplastic particles will have negative effects on the photosynthetic efficiency of symbionts as well as on metabolic rate of the studied foraminifera species. Finally, we tested if the presence of additionally provided particulate food (algae) influences the amount of ingested plastic in a competitive mode.

## Material and methods

### Used plastic particles and studied foraminifera

We used commercial fluorescent PS beads with a diameter of 2 μm (Sigma-Aldrich©, L4530) since they are easy to handle and often used in various biological experiments. Most common plastic polymers have a density between 0.9 and 1.5 g cm^−3^ while seawater has a density of around 1.02–1.03 g cm^−3^^31^. PS is one of the most used plastics and therefore also one of the most frequent microplastics found in the ocean. The particle size was chosen because it overlaps with the particle size range of most foraminiferal foods in the ocean, covering the size range of algae ingested by *A. lobifera*. Particle density is another factor to be considered. PS has a density of 1.05 g cm^−3^ and microplastic particles made of PS therefore slowly sink, to accumulate on the sea floor, which is the habitat of LBFs.

Foraminifera were taken from the main culture collection at the Department of Palaeontology of the University of Vienna. The origin of *A. lobifera* was the bay of Agia Pelagia, Crete, Greece, while *H. depressa* derived from a shark tank from the ‘Haus des Meeres’ in Salzburg, Austria. Both main cultures are run at a temperature of 25 °C and a salinity of 38, with an eight-hour light period over 24 h. We selected adult individuals of approximately the same size containing brownish cytoplasm, which covered the whole test. Both species have diatom-derived symbiotic algae and they were fed with different algae (non-symbiotic species) during the experiments.

### Experimental setup

Experiments were run consecutively for each species. Each experiment was split into three setups and three treatments. One setup was used for pulse amplitude modulated (PAM) fluorescence microscopy, one for stable isotope tracing (SI) and one for the microplastic particle exposure. For all methods setups, measurements were conducted at the start of the experiment and then after 1, 7, 14 and 21 days. Each setup comprised three treatments with six replicates each:

*Treatment C*: Control group; individuals were incubated in pure artificial sea water with a salinity of 35 for *H. depressa* and 38 for *A. lobifera*. Artificial sea water was made from MilliQ water stocked with artificial reef salt (Aquaforest®).

*Treatment P*: Plastic group; individuals were incubated in artificial sea water, under the same conditions as C, but with microplastic particles added. The particles used were yellow-green-fluorescent PS beads with a diameter of 2 μm (Sigma-Aldrich©, L4530). The final concentration was 52.5 mg plastic L^−1^ seawater, which is equivalent to approximately 100,000 particles per ml water. This concentration of PS is artificial high to maximise the effect of PS on the tested organisms.

*Treatment PA*: Plastic and algae group; individuals were incubated in artificial sea water under the same conditions as C, but with both microplastic particles and living microalgae of the species *Nannochloropsis salina* added. *Nannochloropsis* was chosen because of its worldwide occurrence (both foraminifera species can reasonably come across this alga in nature) and its size of around 2 μm in diameter which is congruent with the microplastic particle size.

### Analytical methods

PAM-analysis represents a non-invasive method, not harming the organisms during measurement. After all six individuals (replicates) were measured to assess their initial fluorescence values, crystallisation dishes were prepared like described above—one for each culture treatment (C, P, PA). Six individuals per setup were placed separately per dish. The same individuals were used throughout the whole experiment and placed back in their respective dish after measurements took place. Dishes were sealed with parafilm to guarantee constant culture conditions and to minimize evaporative losses. Analyses were conducted with an Imaging PAM device, which not only allows measuring the quantum yield of the symbionts but also visualising their fluorescent area to locate the exact position of photosymbionts (MICROSCOPY-Version of the M-Series, Hein Walz GmbH; ImagingWin software v 2.56p). Variable quantum yield (Fv/Fm) is the ratio between the variable fluorescence (Fv) and the maximum fluorescence (Fm) and represents the efficiency of photosystem II. Fv/Fm is an indicator of the overall physiological state of photosynthetic organisms. Individuals were picked from the respective crystallisation dish with a fine brush and placed on a glass slide with a small chamber that offered enough space for keeping the organism intact. After 5 min pre-darkening, the mean Fv/Fm of the photosymbionts within the foraminifera was measured and images were taken. These images were later analysed using ImageJ to calculate the total photosynthetic active area via pixel counts.

The experimental setup for stable isotope uptake was identical to the setup for PAM analysis, with the difference being that the heavy stable isotopes ^13^C and ^15^N were added in the form of NaH^13^CO_3_ and ^15^NH_4_Cl to a final concentration of 0.235 mmol ^13^C L^−1^ and 0.220 mmol ^15^N L^−1^, respectively. Individuals were picked from their crystallisation dish after the respective incubation time, dried, and placed into pre-weighted tin capsules (Sn 99.9%, IVA Analysentechnik GmbH & Co. KG). Afterwards the foraminifera were decalcified using 12.5 μl 5% hydrochloric acid and placed in a drying oven at 50 °C for three days to remove all moisture. After weighing to the nearest µg, specimens were stored in a desiccator until isotope measurements were done. The measurements were carried out at the Stable Isotope Laboratory for Environmental Research (SILVER) at the University of Vienna using an isotope ratio mass spectrometer (IRMS, Delta V Advantage with a ConFlo III interface to an elemental analyser EA-Isolink, Thermo Scientific). The ratios of ^13^C/^12^C and ^15^N/^14^N were determined and the amount of incorporated isotopes was calculated as described in Lintner et al.^32^.

For plastic bead ingestion, the setup as outlined above was used. Individuals were picked from the crystallisation dishes with a fine brush, washed and brushed off three times with pure artificial sea water to get rid of any PS particles that might stick externally to the tests. After this cleaning process, individuals were transferred to a glass slide and the calcareous test dissolved using 5% hydrochloric acid (HCl). This step was necessary since otherwise the fluorescence signal could have been quenched through the test structure. Applying the low concentration of HCl for decalcification caused only slow formation of CO_2_ bubbles which kept the organic lining of the organism largely intact. The specimens were then analyzed under a fluorescence microscope (Axio-Imager.M1 ZEISS® equipped with 10-AF-488 filter). Pictures of every individual were taken in the fluorescence mode and in the transmitted light mode (ZEISS ZEN software v 1.0.1.0). Afterwards, the two pictures were compared to determine the exact location of PS particles within the organism. Besides the location of the microplastic particles, the number of incorporated particles was counted as pixel counts (software “ImageJ” v 1.53t). After calibrating the pixel number of single PS beads, all pixels of the corresponding colour spectrum were counted and the number of beads calculated.

### Statistics

We used the software JASP Team Version 0.8.13. For statistical evaluation of PAM fluorescence measurements and microplastic bead uptake, linear mixed model analyses (LMM, likelihood ratio tests) were performed. We included Fv/Fm, photosynthetically active area, or PS bead uptake as dependent variable, and the treatment, and time of exposure as fixed effects variables. Individuals were coded as random effects factors. For stable isotope uptake, generalized linear models (GLM with gaussian distribution, identity link function) were calculated. The respective measurements were coded as dependent variables, and treatment and time of measurement were fixed effects variables. For testing significant group differences of treatments, we used pairwise contrast with Holm’s p-adjustment. Detailed statistical methods and results can be obtained in the supplementary material.

## Results

### Photosynthetic efficiency

**Figure 1:**
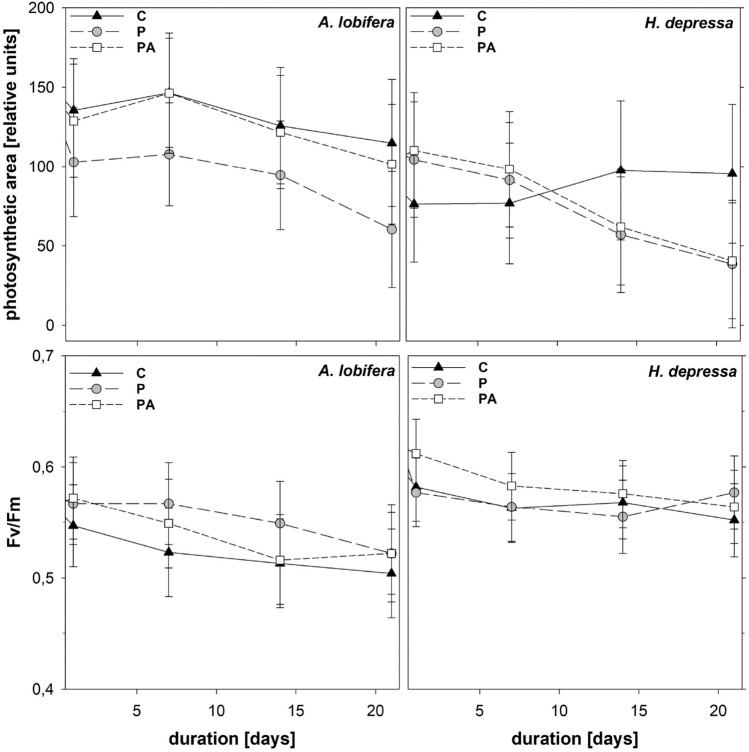
Maximum fluorescence yield Fv/Fm and photosynthetic active area during the experiment for controls C, plastic P and plastic with algae PA. Estimated means with 95% confidence limits (LMM).

Photosynthetic active area differed significantly between species, with *H. depressa* showing smaller areal extents (*p* = 0.002; Fig. 1) but higher Fv/Fm (*p* < 0.001) (Fig. 1).

Overall, Fv/Fm decreased slightly over time, so did photosynthetic active area, except in the controls of *H. depressa* which remained constant. For both P and PA treatments of *H. depressa,* pronounced decreases of photosynthetic active area were observed, which indicates that this species is negatively impacted by the presence of microplastics. The pattern in the experiments with *A. lobifera* was somehow different, which is also reflected by a significant interaction between treatment and time (*p* = 0.004): only treatment P caused a clear area decrease already after the first day of incubation, PA showed a curve shape comparable to the control.

### Stable isotope uptake

**Figure 2:**
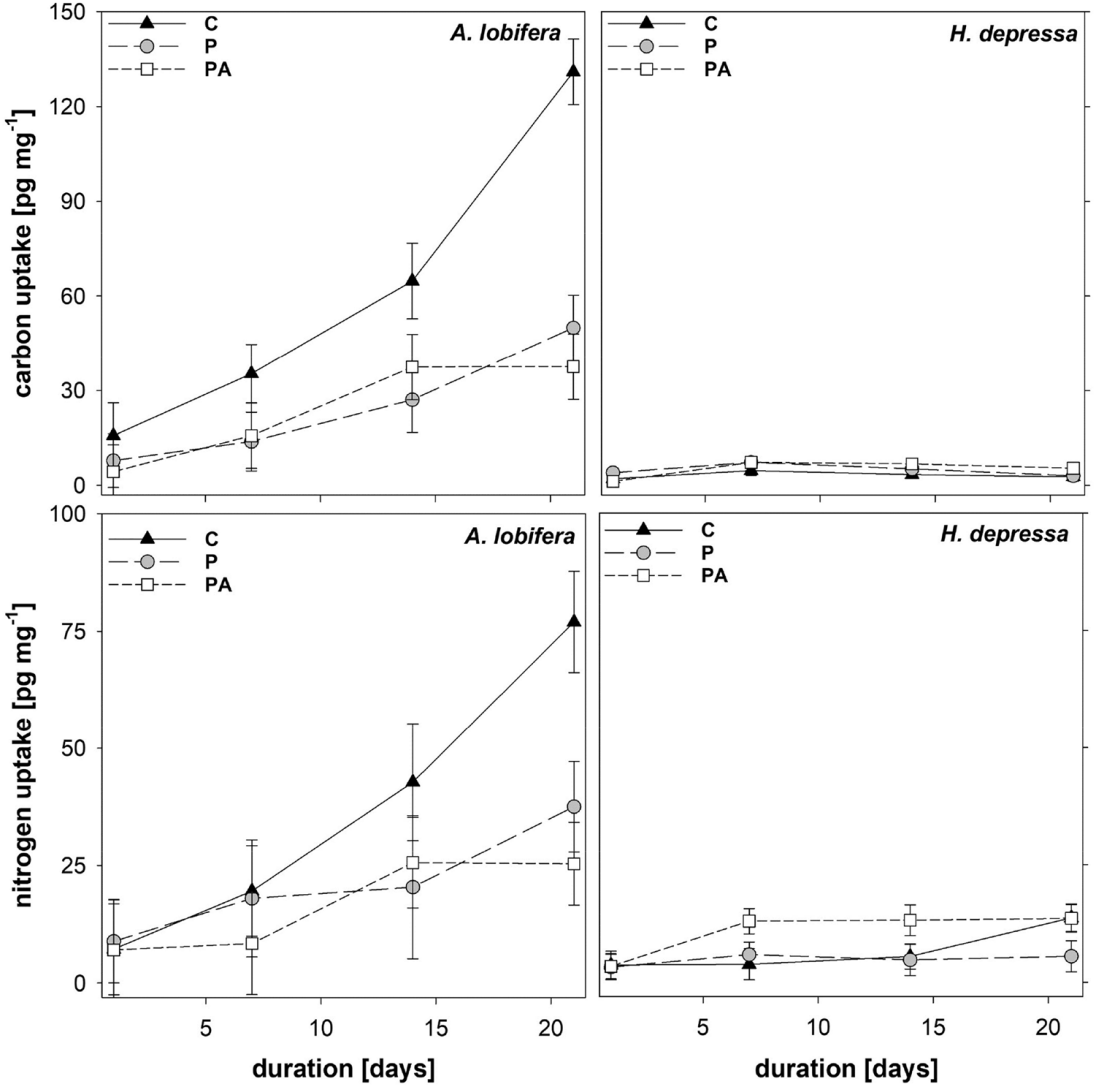
Isotopic uptake of *A. lobifera *and *H. depressa* over time for controls C, plastic P and plastic with algae PA. Estimated means with 95% confidence limits (GLM).

Both the uptake kinetics of carbon (^13^C-bicarbonate) and nitrogen (15N-ammonium) followed highly significant models driven by species, time and treatment (*p* < 0.001, Fig. 2). Compared to *A. lobifera, H. depressa* showed very low incorporation of both elements, which remained almost constant over time (*p* < 0.001). Carbon uptake of the control treatment in *A. lobifera* was significantly higher compared to microplastics-exposed specimens in treatments P and PA (*p* < 0.001). Carbon uptake of P and PA-treated specimens did not differ significantly (*p* = 0.838). Nitrogen uptake showed a similar pattern with significantly higher incorporation in *A. lobifera* compared to *H. depressa*. Although nitrogen uptake in P and PA treatments did not differ in *A. lobifera* but in *H. depressa*. In *H. depressa* nitrogen uptake was stimulated in the PA treatment relative to microplastics only and controls (Fig. 2).

### Microplastics uptake

**Figure 3:**
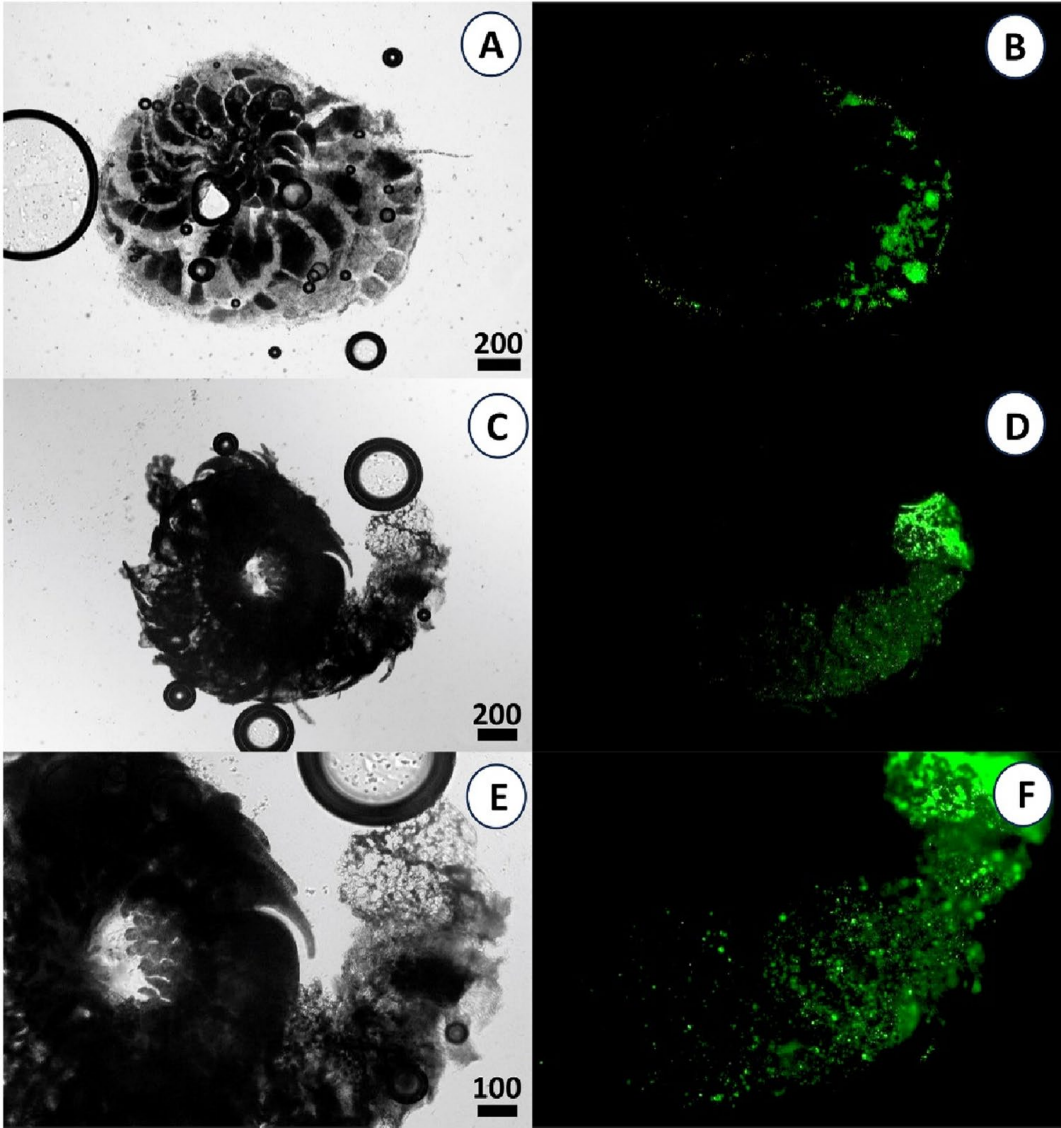
Light microscope (left) and fluorescence microscope (right) images of *H. depressa* (**A**, **B**) and *A. lobifera* (**C**–**F**) after three weeks (21 d) and test dissolution.

Though both foraminifera species showed tight association of tests with microplastic beads, the location of the beads was different for both species. In *H. depressa*, the particles were predominantly found attached to the test surface and were not concentrated around the aperture (see Fig. 3A and B). In *A. lobifera,* microplastic particles stuck to the pseudopods and were concentrated around the aperture of the individuals. However, they were less concentrated towards the older chambers of the foraminifera (Fig. 3C–F).

**Figure 4:**
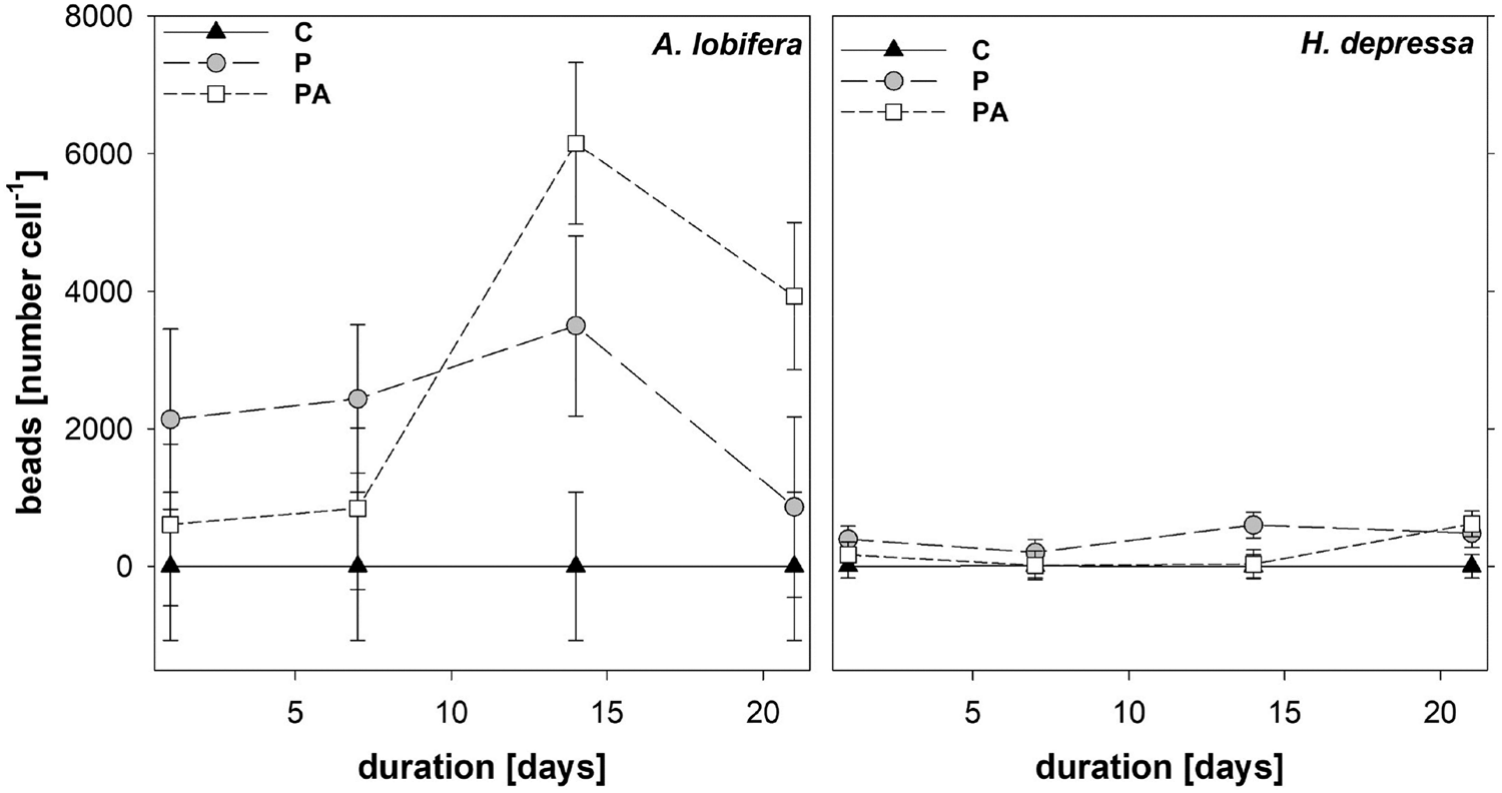
PS beads ingested by *A. lobifera* and *H. depressa* during the experiment for controls (C), and the amendment with microplastic (P) and plastic with algae (PA). Estimated means with 95% confidence limits (LMM).

The ingestion of microplastic beads therefore showed large differences between species, with *A. lobifera* incorporating about 10 × more particles compared to *H. depressa* (*p* < 0.001; Fig. 4). Also time (*p* = 0.004) and treatment (*p* < 0.001) showed pronounced effects in both species. Control treatments (no beads), as expected, showed no detectable ingestion in both species, and though showing some variance mean values were around zero particles per cell over the three weeks. The ingested amount of particles by *H. depressa* differes significantly with treatment (*p* < 0.001) and time (*p* = 0.019). For *A. lobifera* also a significant increase of plastic particles with time (*p* = 0.002) and treatment (*p* < 0.001) was observed. For *A. lobifera*, the presence of algae (PA) suppressed microplastic particle ingestion during the first week, but later (week 2 and 3) promoted microplastic uptake (Fig. 4). Interestingly bead numbers per cell decreased between week 2 and 3, indicating eventual egestion of microplastics.

## Discussion

### Microplastic effects on photosymbiont activity

We applied non-invasive PAM fluorescence imaging to assess possible effects of microplastic particles on foraminifera and particularly on the functionality of their photosymbionts. This PAM approach has been used in previous studies to measure effects of pollutants and environmental parameters like heavy metals, toxic substances, temperature and total alkalinity on foraminiferal symbiont activity^33–35^. From a methodological perspective, PAM imaging primarily indicates the condition of the photoactive symbionts, and thus provides indirect information on the host performance but important insight into holobiont systems biology. If addressing the whole organism (holobiont), complementary approaches are recommended. In the current study, we measured SI uptake to follow inorganic N (ammonium) assimilation via mostly the host cell cytoplasm and bicarbonate assimilation which is predominantly performed via photosynthetic CO_2_ fixation by the symbionts, to complement the data from PAM-analysis. Fv/Fm values of around 0.55 to 0.65 suggest healthy photosymbionts. This means that those photosymbionts that were present (see photosymbiotic active area if photosymbiont numbers change) were not impaired by microplastic presence and uptake. The general and slight, but significant decrease of Fv/Fm during the experiments (*p* < 0.001) indicates a marginal reduction of the overall photosynthetic performance of the symbionts after three weeks of culturing conditions. We did not find a significant influence of plastic bead exposure on photosymbiont activity in *A. lobifera*, which is in accordance of the study of Langlet et al.^28^ investigating respiration (oxygen consumption rates) of *Haynesina germanica* in the presence of PP particles.

However, we found a loss of photosynthetic active area in *A. lobifera* in the presence of microplastics alone (in P not in PA treatment) and in *H. depressa* in any microplastics treatment (P and PA treatments), independent of co-addition of algae. This pattern was also reflected by a significant interaction between treatment and time (*p* = 0.004), indicating a reduction in the number of photosymbionts per cell in both species due to microplastics presence. The response could be seen as intracellular energy generation process to cover the increased energy demand of microplastic-impaired foraminifera, by partial digestion of their own photosymbionts (partly loss of their originally hosted symbionts). *A. lobifera* is dependent on both, on organic metabolites released by symbionts and on the ingestion of solid food such as algal particles to meet their nutritional and energy demands. Based on the isotopic uptake there is almost no difference between P and PA treated foraminifera, which may suggest that plastic is not the limiting factor. As inert microplastic beads can compete for ingestion and uptake mechanisms with food particles in foraminifera, extra energy is generated from digestion of photosymbiont in the situation of reduced or no food particle ingestion under microplastic contamination. Foraminifera encircle their food with a network of pseudopodia that extend from the apertures in the test. For both microplastic (P and PA) treatments of *H. depressa*, which is exclusively depending on the metabolism of their photosymbionts and dissolved nutrient uptake, a pronounced decrease of the photoactive area was observed, which indicates that this species is generally negatively impacted by the presence of microplastics, in absence of any competitive interaction between solid food and plastic particles in the feeding process. A similar behavior was found not only in foraminifera but also in other organisms when they came into contact with microplastics. In both sea anemones and corals it was observed that the number of photosymbionts were reduced by the presence of plastic particles and the host organisms therefore suffered^36^.

### Microplastic ingestion and food uptake

Tracing of SI uptake revealed big differences in dissolved element uptake and assimilation between species, with *A. lobifera* showing about 10 × higher SI incorporation compared to *H. depressa*. *A. lobifera* showed a strong increase in SI assimilation over time, while SI incorporation in *H. depressa* quickly stabilized at very low levels. The constant values of SI incorporation in *H. depressa* are interpreted as reduced metabolic activity at later stages, as in photoactive organisms provided isotopes should accumulate over time. It is obvious that microplastic beads resulted in reduced SI uptake, particularly in *A. lobifera*, which suggests that the microplastic beads have direct inhibitory effects on the foraminiferal host reducing ammonium assimilation and, on the symbionts, inhibiting CO_2_ fixation, though the exact mechanism of this is as yet unknown. Because of their different nutrient foraging strategies and energy generation, the two foraminifera species were also expected to respond very differently to microplastic particles.

Our results clearly indicate that *A. lobifera* ingested microplastic beads, which is in accordance with other existing studies^13,26,27^. Plastic beads and food particles were comparable in size. It is assumed that the main pathway of plastic particles entering foraminifera is shared with food particles^26^. *A. lobifera* stretches out its pseudopodia, mainly extending from its aperture^37^, and uses them to catch food particles for ingestion. Plastic particles that get stuck on the pseudopodia will be transported to the aperture and are ingested by the foraminifera. This can cause competitive interactions, i.e. algal presence reduces microplastic bead ingestion (or otherwise), or positive (facilitation) interactions if algal presence boost feeding activity and thereby increases co-ingestion of food particles and microplastics. In this study these interactions switched along the time axis following the food and microplastics pulse, from competitive during the first week with low feeding activity to positive during week two and three in *A. lobifera*. Joppien et al.^29^ found that the closely related *A. lobifera gibbosa* is able to differentiate between plastic and true food particles, enabling selective feeding. Such selective uptake mechanisms followed by egestion processes of microplastic particles, if ingested, might also explain the pattern seen in Fig. 3 for weeks 2 and 3. Selective uptake, however, come with increased energy costs. *A. lobifera* is able to incorporate food by ingesting particulate food and by taking up and assimilating dissolved nutrients. Microplastic particles therefore can directly interfere with particulate food uptake but not with carrier protein-based uptake of solutes through their plasma membrane. At this point it should be mentioned that the potential effect of leaching of additives from the plastic beats is not examined in this study. These additives could also have a negative effect on the foraminifera and their symbionts, but this is probably negligible considering the short incubation period of only three weeks.

*Heterostegina depressa* on the contrary showed no pronounced algae effect on microplastic particle ingestion. Moreover, this species showed no microplastics effect on inorganic carbon fixation (photosymbionts) while ammonium assimilation (host cells) was stimulated temporally, if triggered by simultaneous presence of microplastics and algae. In part this response is explained by the organism’s behaviour of extending its pseudopodia from narrow test pores^38^ instead of their wide aperture, the test pores being too small for bead intake into the test interior. *H. depressa* does not therefore actively feed on particulate food matter and therefore should not incorporate any particles from its surrounding. Particles of natural or synthetic origin stuck to the pseudopods will eventually end up at the point of origin of the pseudopods and eventually clog the foraminifera’s test pores, yet without interfering with food particle uptake. *H. depressa* fully depends on its photosymbionts and on dissolved nutrient uptake and assimilation. Therefore, effects of microplastics on the nutrition of *H. depressa* were minor here, given the minor response to microplastics in carbon and nitrogen uptake, and chlorophyll fluorescence. However, the loss of photosymbionts as indicated by marked decreases in photoactive area of the symbionts indicates digestion of photosymbionts and negative nutritional effects on this species (Fig. 1).

## Conclusions

We studied photosymbiont activity and element uptake by *H. depressa* and *A. lobifera* in the presence and absence of PS microplastic particles over a period of three weeks. It turned out that plastic microparticles had a measurable negative impact on the photoactive area of *H. depressa*, which is exclusively dependent on symbionts. Together with the constant and low incorporation of dissolved nutrients found in *H. depressa* and the fact that microplastic particles were mainly located on the test surface, we assume other negative effects on their photosymbionts, which minimizes metabolite uptake by the host cells. Simultaneously, the host cells started to digest the photosymbionts, which is reflected in reduced photoactive areas.

For A. *lobifera*, which is an actively feeding protist ingesting algal particles, the photoactive area tended to decrease during exposure to microplastics. We assume that partial digestion of photosymbionts helped to keep the metabolic activity high, especially if no food particles were available. High metabolic activity of host cells and photosymbionts was sustained throughout the experiment, as reflected by the significant accumulation of stable isotopes over time. However, both treatments including microplastic beads caused a significant reduction in dissolved nutrient assimilation thus indicating a severe negative impact of plastic beads on this species. Towards the end of the experiment, the number of plastic particles in A*. lobifera* decreased, which might be interpreted as expelling (egestion) of non-digestible food. This process is energy-consuming, which causes a further reduction of nutrient uptake compared to non-microplastic affected control foraminifera.

### Supplementary Information

Supplementary Information.

## Data Availability

All data generated or analysed during this study are included in this published article and its supplementary information files.
