# Supplementary material for: Effect of micro-plastic particles on coral reef foraminifera
Source: Sci Rep. 2024 May 30;14:12423. doi: 10.1038/s41598-024-63208-3 (PMC11139942; doi:10.1038/s41598-024-63208-3)

**Area Heterostegina (GLMM)**

Summary


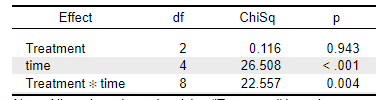


Fit statistics


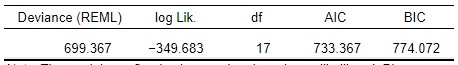


Contrasts (contrast 1 = C/PA, contrast 2 = C/P, contrast 3 = P/PA)


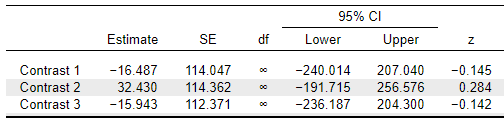


**Yield Heterostegina (GLMM)**

Summary


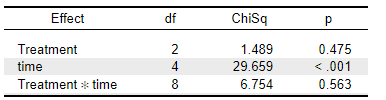


Fit statistics


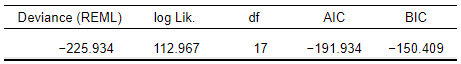


Contrasts (contrast 1 = C/PA, contrast 2 = C/P, contrast 3 = P/PA)


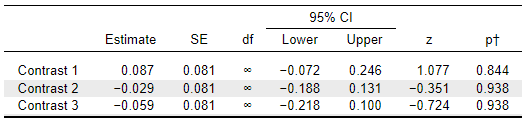


**Area Amphistegina (GLMM)**

Summary


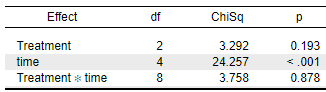


Fit statistics


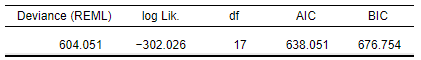


Contrasts (contrast 1 = C/PA, contrast 2 = C/P, contrast 3 = P/PA)


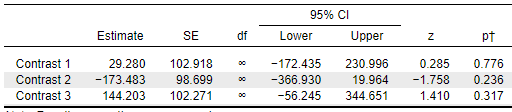


**Yield Amphistegina (GLMM)**

Summary


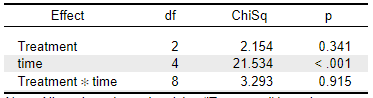


Fit statistics


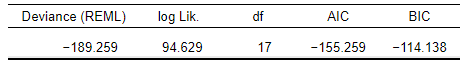


Contrasts (contrast 1 = C/PA, contrast 2 = C/P, contrast 3 = P/PA)


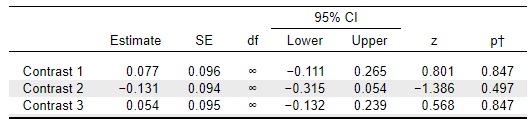


**Carbon Heterostegina (GLM)**

Summary


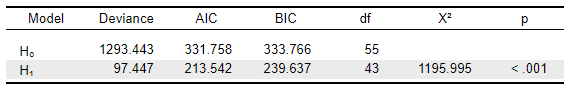


Fit statistics


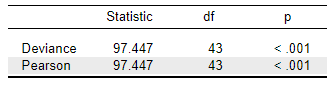


Contrasts (contrast 1 = P/PA, contrast 2 = C/PA, contrast 3 = C/P)


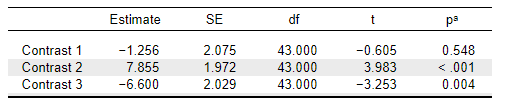


**Nitrogen Heterostegina (GLM)**

Summary


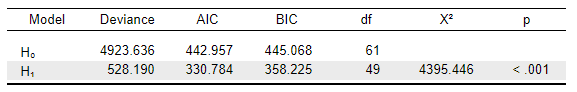


Fit statistics


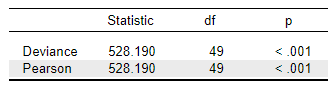


Contrasts (contrast 1 = P/PA, contrast 2 = C/PA, contrast 3 = C/P)


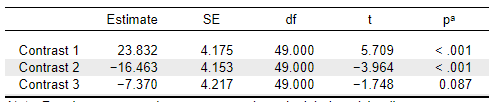


**Carbon Amphistegina (GLM)**

Summary


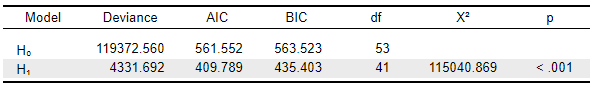


Fit statistics


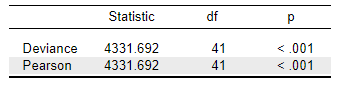


Contrasts (contrast 1 = P/PA, contrast 2 = C/PA, contrast 3 = C/P)


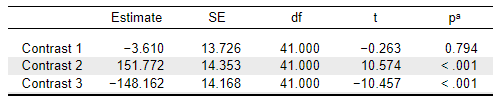


**Nitrogen Amphistegina (GLM)**

Summary


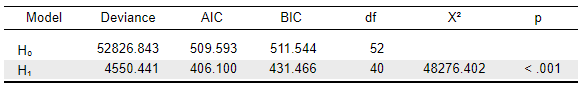


Fit statistics


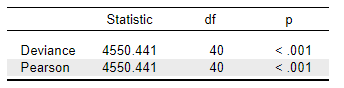


Contrasts (contrast 1 = P/PA, contrast 2 = C/PA, contrast 3 = C/P)


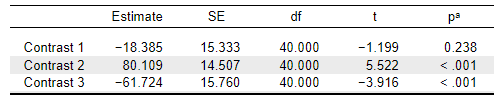


**Beads Heterostegina (GLMM)**

Summary


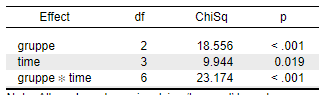


Fit statistics


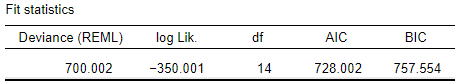


Contrasts (contrast 1 = P/PA, contrast 2 = C/PA, contrast 3 = C/P)


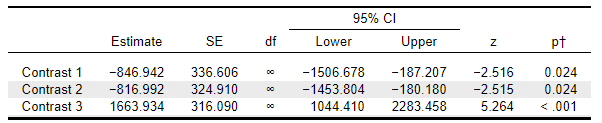


**Beads Amphistegina (GLMM)**

Summary


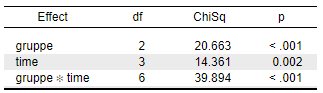


Fit statistics


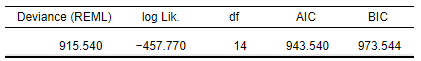


Contrasts (contrast 1 = P/PA, contrast 2 = C/PA, contrast 3 = C/P)


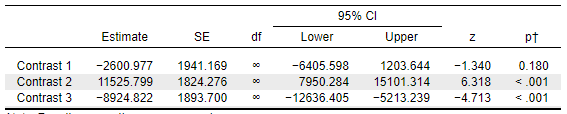

Supplement: Supplementary file 1 — Supplementary Information. [file 41598_2024_63208_MOESM1_ESM.docx]
